# Heart Rate Variability During a Joint Attention Task in Toddlers With Autism Spectrum Disorders

**DOI:** 10.3389/fphys.2018.00467

**Published:** 2018-05-01

**Authors:** Lucia Billeci, Alessandro Tonacci, Antonio Narzisi, Zaira Manigrasso, Maurizio Varanini, Francesca Fulceri, Caterina Lattarulo, Sara Calderoni, Filippo Muratori

**Affiliations:** ^1^Institute of Clinical Physiology, National Research Council of Italy, Pisa, Italy; ^2^IRCCS Stella Maris Foundation, Department of Developmental Neuroscience, Pisa, Italy; ^3^Department of Information Engineering, University of Pisa, Pisa, Italy; ^4^Child Neuropsychiatry Unit, Hospital “Madonna delle Grazie”, Matera, Italy; ^5^Department of Clinical and Experimental Medicine, University of Pisa, Pisa, Italy

**Keywords:** autism spectrum disorders, autonomic nervous system, joint attention, wearable sensors, eye-tracking

## Abstract

**Background:** Autism Spectrum Disorders (ASD) are a heterogeneous group of neurodevelopmental disorders featuring early impairments in social domain, with autonomic nervous system (ANS) unbalance possibly representing a useful marker for such disturbances. Impairments in joint attention (JA) are one of the earliest markers of social deficits in ASD. In this study, we assessed the feasibility of using wearable technologies for characterizing the ANS response in ASD toddlers during the presentation of JA stimuli.

**Methods:** Twenty ASD toddlers and 20 age- and gender-matched typically developed (TD) children were recorded at baseline and during a JA task through an unobtrusive chest strap for electrocardiography (ECG). Specific algorithms for feature extraction, including Heart Rate (HR), Standard Deviation of the Normal-to-Normal Intervals (SDNN), Coefficient of Variation (CV), pNN10 as well as low frequency (LF) and high frequency (HF), were applied to the ECG signal and a statistical comparison between the two groups was performed.

**Results:** As regards the single phases, SDNN (*p* = 0.04) and CV (*p* = 0.021) were increased in ASD at baseline together with increased LF absolute power (*p* = 0.034). Moreover, CV remained higher in ASD during the task (*p* = 0.03). Considering the phase and group interaction, LF increased from baseline to task in TD group (*p* = 0.04) while it decreased in the ASD group (*p* = 0.04).

**Conclusions:** The results of this study indicate the feasibility of characterizing the ANS response in ASD toddlers through a minimally obtrusive tool. Our analysis showed an increased SDNN and CV in toddlers with ASD particularly at baseline compared to TD and lower LF during the task. These findings could suggest the possibility of using the proposed approach for evaluating physiological correlates of JA response in young children with ASD.

## Introduction

Autism spectrum disorders (ASD) are a heterogeneous group of neurodevelopmental conditions featuring impairments in social communication, as well as restricted or stereotyped interests and behaviors (American Psychiatric Association, 2013). Its prevalence is constantly increasing-up to around 1 out of 50 children (Blumberg et al., 2013)-but the cause of such condition is yet unknown, despite being suggested that a gene-environment interaction probably could be at the basis of the disorder (Meek et al., 2013).

Due to the wide clinical and etiological heterogeneity of ASD, the early identification of a set of informative risk markers represents a key-challenge to enable early detection and early diagnosis of ASD (Sacrey et al., 2015), crucial steps for a clinical assessment of this disorder. In fact, early diagnosis allows creating the field for undertaking early specific intervention, in turn bringing in benefits for both the children, even as young as 18-month-old (Rogers et al., 2014; Brian et al., 2015), and the community at large (Schreibman et al., 2015).

Several studies suggest that Autonomic Nervous System (ANS) activity is linked to social functioning in individuals with ASD, suggesting a role of this system in regulating social interactions in this condition (Dawson and Lewy, 1989; Romanczyk and Gillis, 2006; Faja et al., 2013; Sheinkopf et al., 2013; Neuhaus et al., 2014). Social functioning and communication are managed by both sympathetic (SNS) and parasympathetic (PNS) branches of the ANS (Porges, 2001, 2003). The PNS appears to have an important role in social functioning, being partially mediated by the myelinated vagus, in turn innervating several organs in the face and in the neck, and affecting various functions, including cardiac activity, influencing social behavior and communication (Neuhaus et al., 2016). In socially rich settings, an increased PNS activity eases approach to others, as well as adaptive social behavior (Porges, 2001). In addition, social engagement takes advantage of SNS activation, as well. SNS brings to increased heart rate (HR), sweating and alert state, mainly through acting on the so-called “fight or flight” mechanism (Stifter et al., 2011; Diamond and Cribbet, 2013). Summarizing, both SNS and PNS are somewhat involved in social behavior, with SNS activation reflecting a threat-oriented response, while PNS dominance easing adaptive social engagement (Bal et al., 2010).

Several approaches could be employed to assess SNS and PNS, including minimally obtrusive methods based on electrodermal activity (GSR, Galvanic Skin Response) and electrocardiography (ECG). Concerning GSR, it is thought that a reduced electrodermal activity is associated with decreased SNS influence (Hubert et al., 2009), whereas an increase in skin conductance during specific social tasks subtends higher SNS activation (Mathersul et al., 2013). Concerning the ECG signal, the PNS contribution (and, consequently, the corresponding SNS activation level) can be unobtrusively assessed by studying the heart rate variability (HRV) (Porges, 2001) and, more specifically, the contribution to beat-to-beat variability at higher frequency, as well as the respiratory sinus arrhythmia (RSA) (Berntson et al., 1997). RSA, in particular, was seen to predict social responsiveness early during childhood (Patriquin et al., 2014), with higher PNS detected by RSA analysis associated with an increase in social competence and engagement, adaptive coping strategies, and spontaneous eye gazes (Fabes et al., 1993; Eisenberg et al., 1995; Henderson et al., 2004; Heilman et al., 2007). Several works have been conducted in autistic children, highlighting a lower baseline RSA with respect to typically developing (TD) peers (Guy et al., 2014), associated with weaker social and communication skills (Watson et al., 2010; Neuhaus et al., 2016), and significantly altered reactions to social-like stimuli (Van Hecke et al., 2009), which suggest a role of RSA in social reactivity. However, the merging of markers of sympathetic and parasympathetic activation has been rarely assessed in young children with ASD (Neuhaus et al., 2016).

Joint attention (JA), which is defined as the ability to coordinate visual attention with another person and then shift the gaze toward an object or event (Mundy and Gomes, 1998), is one of the more precocious and consistent early signs of ASD (Charman, 2003). Impairment in JA is primarily a social or social-cognitive phenomenon as it implies to look into the eyes of a social partner and to coordinate their visual attention with him (responding JA). Empirical studies suggest that JA in infancy is pivotal for learning, language development, and social-cognitive development in the first years of life (Rothbart and Bates, 1998; Thurm et al., 2007; Mundy and Jarrold, 2010), and is a significant predictor of social competence and cognitive outcomes in TD children as well as those with neurodevelopmental disorders. Thus, measuring behavioral or physiological parameters during a JA task is a marker of social processes in ASD. Several studies have described abnormal social response in subjects with ASD during live JA tasks, as well as eye-tracking (Chawarska et al., 2003; Bedford et al., 2012; Falck-Ytter et al., 2012, 2015; Billeci et al., 2016a) or neuroimaging experimental paradigm (Redcay et al., 2012; Jaime et al., 2016).

Wearable systems and wireless technologies, allowing monitoring patients in an unobtrusive way, are particularly suitable for the recording of physiological parameters in very young children with neuropsychiatric conditions such as ASD during social tasks. Few previous investigations measured HRV in very young children with ASD, with some (Zantinge et al., 2017a,b) did not use wearable devices, possibly increasing discomfort of ASD patients, motion artifacts in the heart rate processing, and consequently to exclusion of some non-analyzable data. To our knowledge only one study (Watson et al., 2012 used wireless ECG sensors, in very young children with ASD, however involving slightly older children than in the present study. Specifically, the authors used the Mini-Logger unit, which was placed in the child's pocket or a waist pack and attached to two surface electrodes placed on the child's chest. Twenty-two children with ASD (26.1 ± 3.3 months), 15 typically developing boys 15 (33.0 ± 6.1 months) 14 typically developing boys (12.9 ± 3.8 months), recruited as a language age comparison group, were assessed with behavioral (looking) and physiological (heart rate and respiratory sinus arrhythmia) measures while looking to nonsocial and child-directed speech stimuli. Although some children were excluded to technical problems or excessive movement, most of the children completed the tasks without complications. The authors found an increased heart rate in children with ASD suggesting that young children with ASD have an elevated arousal level.

Given that literature in this field is still poor, the aims of this work were to test the feasibility of using a wearable chest belt for the monitoring of ECG signal in toddlers with ASD, and to measure the ANS response in a group of toddlers with ASD and neurotypical age- and gender-matched controls during a JA eye-tracking task.

## Materials and methods

### Study population

In the present study, we initially enrolled 46 subjects, equally divided into two groups (ASD and TD children). The ASD group was recruited in three different Institutions: the Autism Unit of IRCCS Stella Maris Foundation of Pisa, the Division of Child Neuropsychiatry of the University Hospital of Messina and the Hospital of Matera. The clinical diagnosis of ASD was established according to the Diagnostic and Statistical Manual of mental disorders-5 criteria (American Psychiatric Association, 2013) and confirmed using algorithm cutoffs on the Autism Diagnostic Observation Schedule-Generic (ADOS-G) (Lord et al., 2012), which was administered by ADOS research reliable examiners. In addition, the parents of the children with ASD completed the Modified Checklist for Autism Toddlers (M-CHAT) (Robins et al., 2001). The exclusion criteria were as follows: (a) neurological syndromes or focal neurological signs; (b) significant sensory impairment; (c) anamnesis of birth asphyxia, premature birth, head injury or epilepsy; (d) use of any psychotropic medication; and (e) potential secondary causes of ASD determined by high-resolution karyotyping, DNA analysis of Fragile-X or screening tests for inborn errors of metabolism.

**Figure 1:**
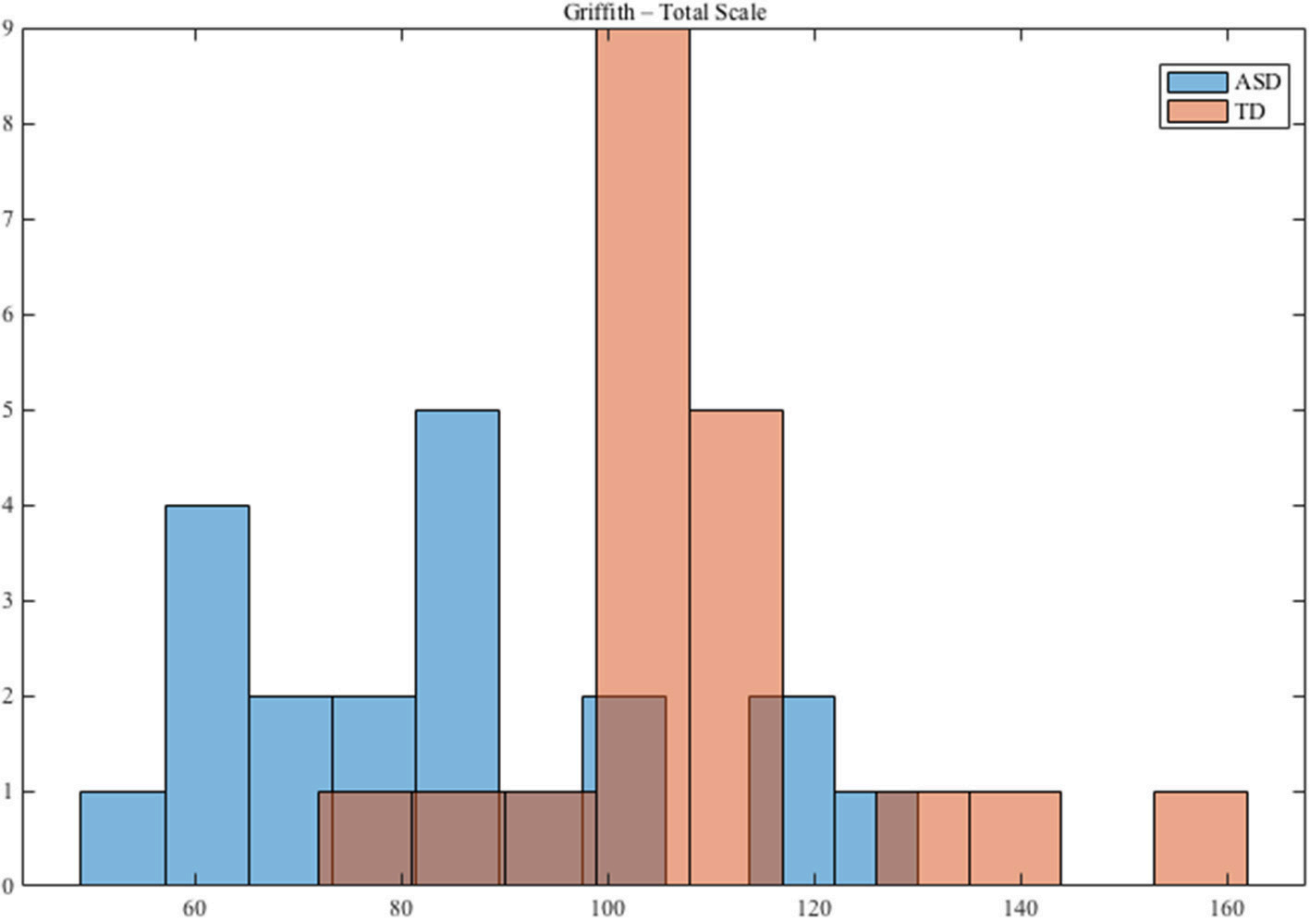
Distribution of Griffith Performance for ASD and TD groups.

The participants with TD were recruited from daycares in the Pisa, Messina and Matera metropolitan areas. All children (ASD and TD) received a nonverbal developmental evaluation through the administration of the performance subscale of the Griffiths Mental Developmental Scales (Griffiths, 1984). Figure 1 shows the distribution of the Griffiths Total Scale for ASD and TD.

The inclusion criteria for TD children were an age between 18 and 36 months and the Child Behavior Checklist 1½−5 (CBCL; Achenbach and Rescorla, 2000) Total score under the borderline/clinical range. Exclusion criteria were family risk for ASD or other neurodevelopmental disorders, visual or auditory impairments and any concerns from the caregivers. After the application of the inclusion and exclusion criteria, the final sample was composed of 40 subjects, equally divided into the two groups (see Results for details). A description of the final study population is displayed in Table 1.

**Table 1:** Study population (significance: *p* < 0.05).

	**ASD**	**TD**	***p*-value**
*N*	20	20	1.00
Age (months, mean ± SD)	26.1 ± 3.3	26.2 ± 3.7	0.48
Gender (M/F)	14/6	15/5	0.80
Griffith Performance (mean ± SD)	81.9 ± 21.8	108.9 ± 19.6	0.001
ADOS-G, module 1–*Total*	14.9 ± 4.5	–	–
ADOS-G, module 1–*Communication*	4.9 ± 2	–	–
ADOS-G, module 1–*Interaction*	9.8 ± 3.4	–	–
ADOS-G, module 1–*Calibrated Severity Score*	7.3 ± 2.1	–	–

An informed consent was obtained from all parents of the children enrolled, after receiving an exhaustive explanation of the study. The experimental procedures and the informed consent were approved by the ethics committee of the IRCCS Stella Maris Foundation (Calambrone, Pisa, Italy). The study protocol conforms to the ethical guidelines of the 1975 Declaration of Helsinki.

### Assessment scales

The ADOS-G (Lord et al., 2012), administered by AN, certified to administer ADOS in clinical and research setting, is an observation measure of current autism symptom severity; on the basis of language level, the module 1 was used for all children. We have also used the Calibrated Severity Scores (CSS), a standardized metric developed to assess autism symptoms as a clinical entity distinct from cognitive and adaptive differences. This metric provides a means to assess symptoms of autism over time in a range between 1 and 10, where 1–3 accounts for no spectrum, 4–5 for ASD, and 6–10 for autism. In the original validation study, these scores were shown to be less influenced by verbal IQ, which accounted for 43% of variance in raw ADOS scores and only 10% in CSS scores (Gotham et al., 2009).

The M-CHAT (Robins et al., 2001) is validated for screening toddlers between 16 and 30 months of age, to assess risk for ASD. The M-CHAT can be administered and scored as part of a well-child check-up, and can be also used by specialists or other professionals to assess the risk for ASD. The primary goal of the M-CHAT was to maximize sensitivity, meaning to detect as many cases of ASD as possible.

The Griffiths Mental Developmental Scales (Griffiths, 1984), administered by expert clinicians, are a standardized developmental test for children from birth to 96 months of age. They comprise six scales, but because of the young age of the children, only five out of the six were administered: Locomotor, Personal-Social, Language, Eye and Hand Coordination, and Performance. Raw scores have been computed for each subscale and converted to general quotient scores, using tables of the analysis manual.

The CBCL (Achenbach and Rescorla, 2000) is a 100 item parent-report measure designed to record the behavioral peculiarities of preschoolers. Each item describes a specific behavior, and the parent is asked to rate its frequency on a three-point Likert scale. The scoring gives, among others, three main scores (Internalizing, Externalizing, and Total Problems). A T-score (for Internalizing and Externalizing, and for Total Problems) of 63 and above is considered clinically significant, and values between 60 and 63 identify a borderline clinical range; values beneath 60 are considered not clinical.

### Acquisition protocol

Electrocardiography (ECG) signals were recorded during an eye-tracking JA task previously described by our group (Billeci et al., 2016a). Toddlers' gaze was recorded by means of the SMI 500 Eye Tracking device provided by SensoMotoric Instruments (Teltow, Germany), while video stimuli were presented on a 22-inch flat monitor. The toddlers sat on a chair seat to limit movements. Simultaneously, ECG signals were wirelessly acquired.

Attention to the stimuli was assured by the simultaneous acquisition of eye-tracking and ECG data. Trials for which the children did not look at the screen were excluded from the analysis and children with more than 50% of excluded trials were eliminated from the final sample. Moreover, all the sessions were recorded with a webcam connected to the stimulus PC.

Briefly, the task consisted in watching some short videos reproducing three JA conditions: a response to JA (RJA) and two initiation of JA (IJA) conditions. The RJA condition consisted of a woman placed between two identical objects, in turn placed in front and on either side of her; she smiled and turned her head toward one of the two objects. On the other hand, in the two IJA tasks, the woman maintained direct gaze but in one case one of the objects activated unexpectedly, while in the other one the object appeared from one end of the frame and crossed the scene. The video sequence lasted about 8 s and was repeated several times, so that the total duration of the task was about 5 min (Billeci et al., 2016a). A total of 12 trials was presented to each child.

The acquisition protocol also included a “baseline” phase of 5 min before the beginning of the task, in which the child was sitting on a chair near to the therapist, without any particular annoyance due to the clinical scenario.

### ECG monitoring

Electrocardiography (ECG) signals were recorded with a smart sensor of the CE certified Shimmer® platform (Burns et al., 2010), composed of two separate boards, one (base-board) that is similar to all the sensors of this factory, and that is programmed by using specifically designed codes, and the second (front-end board) that is able to perform a pre-filtering of the signal acquired reducing the artifacts of the signal. Overall, the ECG module is extremely small (50 × 25 × 23 mm) and light-weight (30 g).

The ECG sensor was modified by adding two pins in order to allow its interfacing with the common Polar™ cardio-fitness chest strap, featuring two dry electrodes in the inner side of the strap, interfaced to the skin surface of the subject for a single-lead acquisition. The chest strap employed is extremely customizable, allowing for monitoring physiological signals in children with different anatomical characteristics, such as the chest diameter. The signals were acquired with a sampling rate of 200 Hz and an A/D resolution of 12 bits. Home-made Matlab™ scripts were then used for ECG pre-processing and feature extraction.

### ECG pre-processing

A stepwise filtering was initially applied to remove artifacts and interferences. Body movements and respiration were removed with a cubic spline 3rd order interpolation between the fiducial isoelectric points of the ECG. A notch filter was also applied to remove the power line interference at 50 Hz and an IIR low pass filter at 40 Hz was applied to eliminate muscular noise. In addition, an interpolation using the Fourier method was applied to the signal to improve RR fiducial points recognition (Task Force of the European Society of Cardiology and the North American Society of Pacing and Electrophysiology, 1996). The Pan-Tompkins method was then adopted to detect the QRS complexes (Pan and Tompkins, 1985), from which the RR series was extracted. Residual artifacts and outliers were removed from RR series by visual inspection. Outliers were replaced by division or summation. The division was applied when the outlier was determined by a failure to detect an R-peak while summation was applied when it was caused by faulty detections of two or more peaks within a period representing the RR interval (RRI).

### Feature extraction

RR series were further analyzed using home-made Matlab™ scripts to extract several time- and frequency-domain features both from the baseline and the task phases.

#### Time-domain analysis

The following time-domain features were extracted and used for analysis:

– Heart rate (HR), expressed as beats per minute (bpm).
– Standard deviation of NN intervals (SDNN), which is a measure of both sympathetic and parasympathetic activity and therefore provides an index of total HRV (Task Force of the European Society of Cardiology and the North American Society of Pacing and Electrophysiology, 1996).
– The coefficient of variation (CV) of the time interval between two consecutive R-waves (RRI) calculated by dividing the standard deviation of RRI (SDNN) by the mean of RRI. We corrected the SDNN with respect to the mean of RRI as HRV and HR are mathematically associated (Sacha and Pluta, 2008; Sacha, 2013).
– pNN10 defined as the percentage of successive normal IBI > × > 10 ms and assessing parasympathetic activity (Mietus et al., 2002).

#### Frequency-domain analysis

The power spectral density was estimated by the Welch method (Welch, 1967), through which we extracted the following frequency-domain features (Task Force of the European Society of Cardiology and the North American Society of Pacing and Electrophysiology, 1996):

– Low Frequency (LF) which is the absolute value of the Low Frequency (LF) power (0.04–0.24 Hz) and emphasizes changes in sympathetic regulation.
– High Frequency (HF) which is the absolute value of the High Frequency (HF) power (0.24–1.04 Hz). This frequency band corresponds to band of the spontaneous breathing frequency of children (i.e., from 0.24 to 1.04 Hz or approximately 15–60 breaths per minute) (Bar-Haim et al., 2000; Heilman et al., 2007; Patriquin et al., 2014). Thus, this band in children overlaps with Respiratory Sinus Arrhythmia (RSA). This measure is mostly related to parasympathetic regulation.
– Normalized Low Frequency (nLF), which is the ratio between LF and the sum of LF and HF.
– Normalized High Frequency (nHF), defined as the ratio between HF and the sum of LF and HF.
– LF/HF Ratio, representing the ratio between the power of LF and HF bands. Its measure indicates the overall balance between sympathetic and parasympathetic systems.

The frequency band limits of LF and HF were selected according to the recommendations for reporting HRV in children and infants (Quintana et al., 2016).

### Statistical analysis

Statistics was performed using SPSS 23 software (SPSS Inc., Chicago, IL, USA). The Shapiro-Wilk test was applied to evaluate whether the variables considered were normally distributed. A repeated-measures ANCOVA was performed with “phase” (i.e., baseline or JA task) as a “within-group” and “group” (i.e., ASD or TD) as a “between groups” factor. When the variables had a non-normal distribution, variables and covariate were transformed in ranks and the analysis of covariance on ranks was performed. Given that the two groups were different in terms of Griffiths performance, this measure was used as covariate in the ANCOVA. In case of a significant phase or phase x group effect (*p* < 0.05), a *post-hoc* analysis was performed to compare differences between phases within each of the two groups (paired-sample *t*-test or Wilcoxon test, according to the distribution of variables) and differences between the two groups (*t*-test or Mann-Whitney test, according to the distribution of variables). Given the exploratory nature of this study, we also reported differences with significance between the two groups within the different phases for all the measures as resulted from independent samples tests (*t*-test or Mann Whitney test, according to the distribution of variables). Statistical correlations between autonomic parameters and selected items from ADOS-G (pointing; response to JA, gesturing, showing, initiation joint attention and unusual eye contact) and Griffith Scales were performed using the Pearson or Spearman Correlation Test.

## Results

### Participants characteristics

After the exclusion criteria application, 3 TD were excluded because the CBCL Total score was over the threshold. Additional 3 ASD children were excluded, one of which due to premature birth, and the other two due to the use of psychotropic drugs.

Thus, the final sample included into the statistical analysis, was constituted of 40 children, 20 TD and 20 ASD.

### Feasibility assessment

The first analysis aimed at assessing feasibility of the approach proposed in monitoring toddlers with ASD and TD controls. The tolerability and comfort of toddlers were judged by a psychologist who was present during the sessions and was further confirmed by the inspection of the videos recorded by the webcam. These observations showed that the system did not cause any kind of annoyance and all 40 children successfully accomplished the experimental protocol proposed without showing sensory-motor and/or behavioral issues in wearing the devices and without any difficulties or constraints. Excessive movements were limited also because in the protocol the toddlers were seated in a chair while watching the stimuli and thus relatively restrained in their physical activity. Both the ASD and the TD group attended the videos at the same way for an acceptable time. Indeed, repeated-measures analysis of variance revealed that there was no significant effect of task or group × task on the number of usable trials (Billeci et al., 2016a). The mean number of usable trials was 11.7 ± 1.2 for the ASD group and 11.2 ± 1.5 for the TD group.

### ECG signal analysis

The second analysis aimed at comparing the two study cohorts according to the features extracted from the ECG signal.

According to the Shapiro-Wilk test, LF and HF (both during baseline and task) had a non-normal distribution while the other variables were distributed normally. Thus, we performed parametric or non-parametric tests according to the variables distribution.

**Figure 2:**
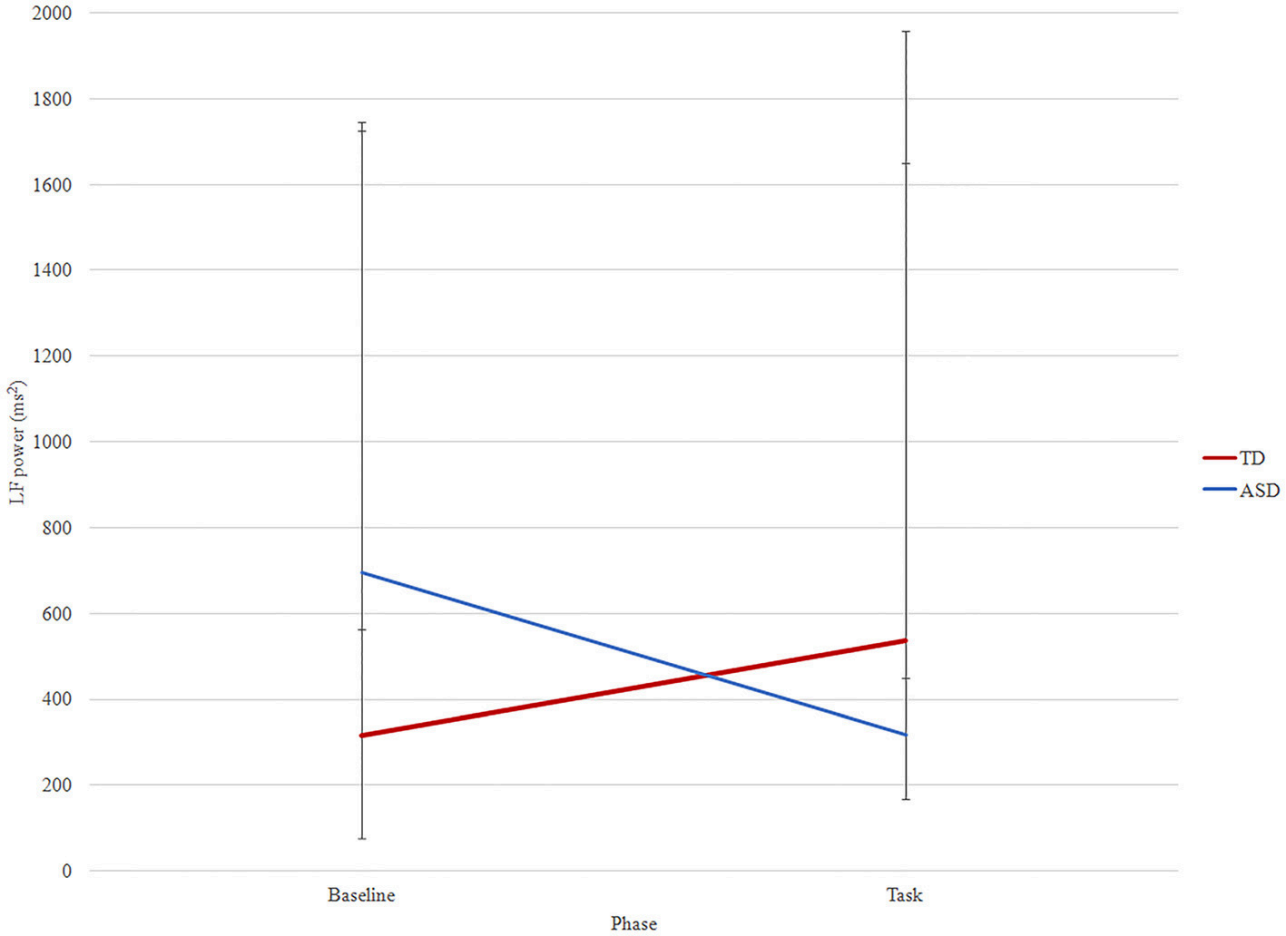
LF changes between phases (baseline and task) in ASD (blue) and TD (red) groups. Median and interquartile are reported.

There was a significant effect of phase x group for LF (*F* = 5.54, *p* = 0.026) (Figure 2). In TD group, LF increased from baseline to task (median ± IQR: 316.00 ± 1162.00 vs. 537.00 ± 3339.50, *p* = 0.04) while in the ASD group it decreased (median ± IQR: 774.00 ± 6601.00 vs. 357.00 ± 1081.60, *p* = 0.04). There was not any significant phase or phase × group interaction for the other time and frequency measures.

Comparing the two groups, we observed that SDNN, CV, and LF were significantly higher in the ASD group compared to the TD group at baseline. In addition, CV was also higher in in the ASD group during the task. All the other time- and frequency-domain features did not differ between-groups, nor within-group. In Table 2 we reported values of all the features for the two groups and the significance obtained independent samples tests.

**Table 2:** Comparison between the *two* study groups in all the single ECG features extracted in the single testing phases (significance: *p* < 0.05).

	**ASD**	**TD**	***p*-value**
**BASELINE**			
HR (bpm, mean ± SD)	104.24 ± 36.41	112.49 ± 22.82	0.41
SDNN (ms, mean ± SD)	0.45 ± 0.63	0.14 ± 0.16	0.04
CV (n.u., mean ± SD)	0.78 ± 0.80	0.28 ± 0.32	0.021
pNN10 (%, mean ± SD)	74.21 ± 23.53	68.57 ± 24.57	0.49
LFn (n.u., mean ± SD)	0.43 ± 0.19	0.38 ± 0.16	0.59
HFn (n.u., mean ± SD)	0.57 ± 0.10	0.62 ± 0.08	0.95
LF/HF Ratio (mean ± SD)	3.81 ± 1.91	4.67 ± 2.04	0.26
LF [ms^2^, median (IQR)]	696.00 (1669.00)	316.00 (1162.00)	0.047
HF [ms^2^, median (IQR)]	1550.00 (3746.00)	344.50 (2587.00)	0.34
**TASK**			
HR (bpm, mean ± SD)	119.06 ± 43.53	120.56 ± 25.43	0.90
SDNN (ms, mean ± SD)	0.26 ± 0.32	0.15 ± 0.22	0.03
CV (n.u., mean ± SD)	0.53 ± 0.44	0.30 ± 0.35	0.09
pNNx (%, mean ± SD)	75.24 ± 19.55	68.01 ± 19.66	0.28
LFn (n.u., mean ± SD)	0.32 ± 0.21	0.41 ± 0.19	0.24
HFn (n.u., mean ± SD)	0.68 ± 0.44	0.59 ± 0.19	0.24
LF/HF Ratio (mean ± SD)	2.86 ± 2.12	3.33 ± 1.91	0.50
LF [ms^2^, median (IQR)]	318.00 (1669.0)	537.00 (1199.30)	0.50
HF [ms^2^, median (IQR)]	1300.00 (2738.00)	472.50 (3110.50)	0.91

*For variables with normal distribution mean and standard deviation (SD) is reported, while for variables with non-normal distributions median and interquartile range (IQR) is reported. n.u.: normalized units*.

### Correlation between RR features and clinical measurements

Concerning the correlation analysis, in the ASD group, CV at baseline was positively correlated with the item “initiation joint attention” of the ADOS-G (*r* = 0.506, *p* = 0.032).

In the TD group, a significant negative correlation between LF at task and Internalizing item of the CBCL Scale (*r* = −0.493, *p* = 0.046) was observed.

## Discussion

The first aim of our study was to test the feasibility of using wireless and wearable technology in toddlers with ASD and TD to record ECG signals, since these technologies are scarcely applied in these populations. The results of our study provided evidence of the feasibility of using a wearable unobtrusive chest strap for assessing ANS activation through ECG signal recording and analysis in such a sample. This could be particularly important in young age as it could provide important physiological markers of social functioning in toddlers with ASD, thus contributing to the diagnosis and the identification of the best therapeutic approach. The system proposed was already successfully applied by our group in schoolers with ASD (Billeci et al., 2016b; Di Palma et al., 2017), thus this study extends our previous results also to toddlers, therefore possibly representing the basis for future related investigations. Moreover, we showed the feasibility of simultaneous recording of eye-tracking and ECG during a social task, like JA, providing the possibility of measuring at the same time gaze pattern and physiological response. These two facts, taken together, foster the employability of this solution for monitoring HR/HRV also in naturalistic, less structured settings, and with particularly fragile populations, including toddlers with early evidence of neurodevelopmental disorders, strengthening the importance of the present solution.

The second aim of the study was to evaluate specific differences in ANS response between toddlers with ASD and typical peers during baseline and, in particular, in response to a JA task. Despite the relatively small sample size of the population enrolled in this research, autonomic dysregulation can be seen among ASD subjects. Indeed, both SDNN and CV (normalized SDNN) were higher in ASD at baseline, indicating an increased HRV at rest, before the start of the task. In fact, SDNN is an index of HRV, indicating the variability of the HR during the whole duration of recording. Indeed, SDNN is mathematically equal to total power of spectral analysis, and so reflects all the cyclic components responsible for variability in the period of recording (Task Force of the European Society of Cardiology and the North American Society of Pacing and Electrophysiology, 1996). Consistently, both absolute LF and HF power, were higher at baseline in ASD, fostering the hypothesis toward an autonomic dysregulation of this population, already very early in childhood, and independently from the task. Thus, the higher SDNN and CV experienced by the children with ASD at baseline could suggest an abnormal global dysfunction of the ASN. Indeed, previous literature suggests that a higher HRV is not always an index of better functioning (Stein et al., 2005). The negative role of excessive high CV at baseline is confirmed by its positive correlation with the item “initiation joint attention” of the ADOS-G, which means that higher problems with the initiation joint attention behavior are correlated with higher CV at baseline. Notably, SDNN (but not CV) remains higher in ASD children compared with TD, during task meaning that the global dysfunction of ANS in these patients persists throughout the recording.

Literature regarding the activation of the ANS in ASD is somewhat inconsistent with some studies indicated an abnormally higher sympathetic, parasympathetic activity or finding no differences with TD (Klusek et al., 2015). In our study, the fact that the between-groups difference in LF is significant while HF is not, may suggest a prevalence of LF component. The higher LF experienced by the children with ASD at baseline could suggest a higher activation of the SNS, related to a somewhat higher anxiety state before the beginning of the task. This fact, indirectly consistent with the existing literature on this topic (Ming et al., 2005; Bal et al., 2010; Daluwatte et al., 2013; Porges et al., 2013; Kushki et al., 2014; Panju et al., 2015).

Focusing on the LF power, it is worth noting that the trend from baseline to task is significantly different between the two groups. Indeed, while in ASD subjects the LF power decreased from baseline to task, TD children displayed the opposite trend, with an increased value of LF during the task. This fact suggests a significantly increased activation of the SNS among TD children during JA, normally concerned with mental effort in attention-demanding tasks (see, for example, Beauchaine et al., 2001). Previous studies have shown that while TD subjects demonstrate increased sympathetic arousal during active tasks, individuals with ASD may not show this effect and could even display paradoxically increased parasympathetic activity compared with controls (Toichi and Kamio, 2003).

As a result, during the task, there is a higher LF in TD than in ASD although it does not reach significance. The positive role of increasing LF during the task, is confirmed by the negative correlation between LF during this phase and Internalizing item of the CBCL Scale I TD group.

In this research, however, RSA does not appear to be different between the two groups, nor between the two phases. RSA has been theoretically linked to social engagement (Porges et al., 2013), and are normally higher in TD children with respect to ASDs (Watson et al., 2012); however, our subjects, possibly due to the extremely young age or to the relatively small sample size, did not display any statistical difference. In general terms, RSA is an index of both respiratory signal and, under very specific conditions may sometimes partially reflect, or be a marker of, cardiac vagal tone (Grossman and Kollai, 2003).

According to Porges (2007), a greater basal RSA amplitude and task dependent RSA suppression, may be a protective factor reducing the risk of developing psychopathologies, particularly those associated with social behavior. However, the results obtained in the present work failed to demonstrate RSA variations either between groups and between phases, fostering the need for further investigations on larger cohorts.

Few studies have investigated changes in HRV in response to social events and stimuli in young children with ASD. Corona et al. (1998) reported that 3–5-year-old children with ASD failed to show an HR decrease to the feigned distress of an examiner, as compared to children with developmental delays (DD). The same group reported that both young children with ASD and a matched group of children with DD (mean age: 4 years) showed decreased HR when viewing a video of babies laughing or crying, suggesting that the children with ASD were as attentive to the videotapes of the infants as was the DD group (Sigman et al., 2003). More recently, Watson et al. (2012) compared physiological responses of children with ASD (mean age: 35 months) to those of chronological age-matched typically developing children (mean age: 33 months) children during exposure to non-social and child-directed speech (CDS) stimuli. They found an increased HR in children with ASD compared to TD, even during periods of sustained looking at stimuli, in both non-social and CDS stimuli. This was interpreted as an overactive sympathetic system or underactive parasympathetic system, or both. The RSA was not different between groups during the task, similarly to our findings; however, the authors did not evaluate the changes compared to the baseline. In another study, the RSA of children with ASD and TD (age range: 2–6 years) was evaluated during a baseline period and a stranger approach paradigm. This study revealed differences in patterns of RSA response to social events in the two groups. Specifically, while children with TD showed a RSA response (decrease from baseline to task) in all the condition, children with ASD were more likely to show a response during the intrusive “proximal” stranger approach than during the initial and less intrusive entry of the stranger into the room (Sheinkopf et al., 2013). Given that RSA reflects social response and engagement with the environment, this finding suggests that a higher level of interaction is needed to elicit normative physiological responses in children with ASD. These results suggest that the ability to modulate cardiac activity of young children in response to a social task is very stimulus-specific. The partial discrepancies between the different studies, including our, indicate that experimental design, and in particular, the intensity, salience, or type of the stimuli can influence ANS response and between-subject differences. In our study, the ASD children, seem not to engage in the task as they do not show an increase of sympathetic activity as the comparison group. However, the RSA did not significantly change from baseline to task in both groups.

Considering our preliminary results and the literature evidences, we could hypothesize that, aside the autonomic dysregulation noticed already at baseline, the ASD subjects did not display the increased SNS activation during the task seen among TD children, demonstrating a lower degree of mental engagement during JA.

Taken together, these results suggest that the unobtrusive measurement of ANS response during JA task, which was seen to be feasible and well tolerated also by ASD toddlers, could represent an early marker of social dysfunction in ASD, contributing to a more objective diagnosis and to the definition of a more tailored treatment protocol. However, as above mentioned, the procedure here described could probably form the basis for future investigations on this specific population in this field.

### Limitations and future developments

Some limitations need to be considered when interpreting the results of this study. First, the relatively small sample size should also be acknowledged, limiting the significance of the results we found. However, we should mention that the restricted age range of toddlers with ASD does not ease the recruitment of a numerous cohort. Second, we did not include a control group with a developmental quotient similar to that of the ASD group. The comparison with a TD group could prevent our result to be considered specific of ASD, and could be questioned whether the group effects reflect differences specifically due to ASD. Nevertheless, this limitation is reduced by the use of the nonverbal development quotient as a covariate in all between-subject comparisons. Finally, we evaluated ANS in response to videos made up of JA tasks, providing a partial assessment of JA as compared with a real-life situation; therefore, also the physiological response could be altered.

In the future, the findings of the study need to be replicated in a larger sample to prove the efficacy of the approach and to consolidate the results obtained. Larger samples will also allow for the evaluation of how different could be the physiological response of subgroups of children with ASD, i.e., high and low functioning children. As JA is an early marker of impairments in ASD, in a larger sample and even in young children it could be important to test the predictive value of ANS assessment for clinical outcomes in these groups. In addition, future studies could include multiple measures of the ANS, including additional HRV features (as the estimation of phasic contribution), GSR and respiration, using synchronized wearable sensors to evaluate relationships within and between components, and their relationships to social response during JA tasks.

## Conclusions

In conclusion, in this study, we demonstrated the feasibility of using a wearable non-invasive technology for characterizing ANS response in toddlers with ASD during a social attention task. Our results possibly suggest autonomic dysregulation in ASD already at baseline. In addition, TD children showed an increased LF during the JA task, with an opposite trend with respect to ASD children. This result possibly demonstrates a different attitude toward JA, with a significantly higher mental effort performed by neurotypical children.

Both ASD and TD subjects, however, fail to exhibit a variation in RSA during JA, proving the necessity of future studies on larger cohorts. The results of this study foster the application of the proposed approach for evaluating physiological correlates of JA response in very young children and toddlers with ASD.

## Author contributions

LB helped in data collection, guided in data and statistical analyses, and wrote the final version of the manuscript; AT performed the data and statistical analysis and made a manuscript draft; ZM helped in the data and statistical analysis; MV contributed in the ECG signals analysis; LB, AN, and FM conceived the study; AN and CL participated in data collection; AN, CL, SC, and FF participated in the clinical assessment of the subjects; FM was responsible of recruitment and diagnosis of children; FM, MV, and CL contributed in the discussion and approval of the paper.

### Conflict of interest statement

The authors declare that the research was conducted in the absence of any commercial or financial relationships that could be construed as a potential conflict of interest.
